# Polyamine Metabolism as a Therapeutic Target in Hedgehog-Driven Basal Cell Carcinoma and Medulloblastoma

**DOI:** 10.3390/cells8020150

**Published:** 2019-02-11

**Authors:** Sonia Coni, Laura Di Magno, Silvia Maria Serrao, Yuta Kanamori, Enzo Agostinelli, Gianluca Canettieri

**Affiliations:** 1 Department of Molecular Medicine, Sapienza University, 00161 Rome, Italy; sonia.coni@uniroma1.it (S.C.); silviamaria.serrao@uniroma1.it (S.M.S.); 2 Center for Life Nano Science@Sapienza, Istituto Italiano di Tecnologia, 00161 Rome, Italy; Laura.DiMagno@iit.it; 3 Department of Biochemical Sciences ‘A. Rossi Fanelli’, Sapienza University, 00185 Rome, Italy; yuta.kanamori@uniroma1.it; 4 International Polyamines Foundation—ONLUS—Via del Forte Tiburtino, 98, 00159 Rome, Italy; 5 Pasteur Laboratory, Department of Molecular Medicine, Sapienza University, 00161 Rome, Italy

**Keywords:** Hedgehog, polyamines, medulloblastoma, basal cell carcinoma, d,l-alpha-difluoromethylornithine

## Abstract

Hedgehog (Hh) signaling is a critical developmental regulator and its aberrant activation, due to somatic or germline mutations of genes encoding pathway components, causes Basal Cell Carcinoma (BCC) and medulloblastoma (MB). A growing effort has been devoted at the identification of druggable vulnerabilities of the Hedgehog signaling, leading to the identification of various compounds with variable efficacy and/or safety. Emerging evidence shows that an aberrant polyamine metabolism is a hallmark of Hh-dependent tumors and that its pharmacological inhibition elicits relevant therapeutic effects in clinical or preclinical models of BCC and MB. We discuss here the current knowledge of polyamine metabolism, its role in cancer and the available targeting strategies. We review the literature about the connection between polyamines and the Hedgehog signaling, and the potential therapeutic benefit of targeting polyamine metabolism in two malignancies where Hh pathways play a well-established role: BCC and MB.

## 1. Polyamines: Metabolism, Mechanism of Action and Role in Tumorigenesis

**Figure 1:**
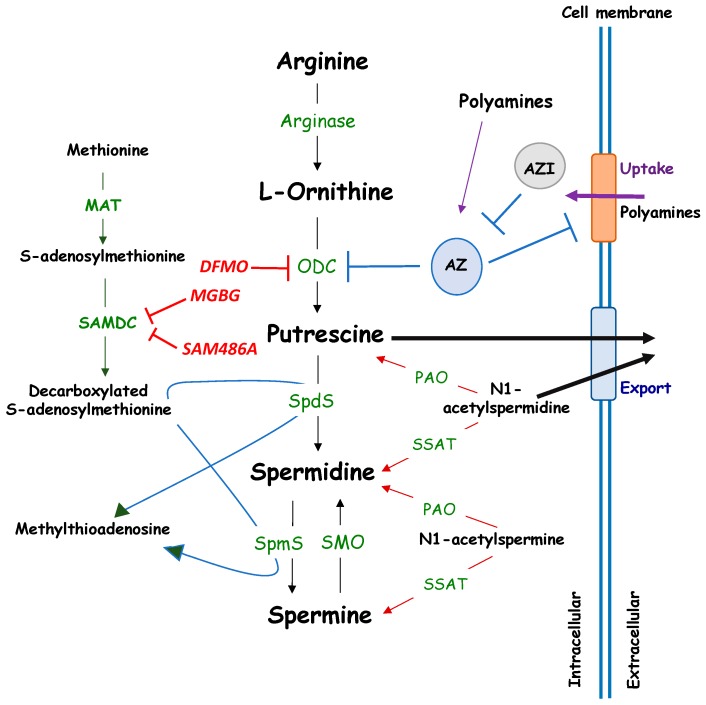
Metabolism of polyamines. MAT: Methionine adenosyl-transferase; ODC: Ornithine decarboxylase; AZ: Antizyme; AZI: Antizyme Inhibitor; PAO: Polyamine oxidase; SAMDC: *S*-Adenosyl-l-Methionine Decarboxylase; SMO: Spermine oxidase; SSAT: Spermidine/Spermine-*N*(1)-acetyltransferase; SpdS: Spermidine Synthase; SpmS: Spermine Synthase. Pharmacological inhibitors of ODC (DFMO) and SAMDC (MGBG, SAM486A) are indicated in red.

Polyamines are ubiquitous, small, positively charged polycations required for both eukaryotic and prokaryotic cellular growth and differentiation. They are low molecular weight, water soluble, aliphatic amines, with pK values between 8.3 and 10.9. Polyamines bind negatively charged molecules under physiological conditions, including DNA, RNA, ATP, proteins or phospholipids [1,2] and are important regulators of various physiological processes. The naturally occurring polyamines spermine and spermidine are the biosynthetic products of putrescine, which is derived from ornithine by the action of ornithine decarboxylase (ODC), one rate-limiting enzyme, together with S-adenosyl-methionine decarboxylase [3] (SAMDC) [4] (Figure 1).

During cancer cell growth, the polyamine biosynthetic pathway is very active and polyamine content is increased in tumor cells and tissues, such as breast, colon, skin and prostate cancers [5]. The elevated polyamine content is often linked to increased putrescine synthesis by ornithine decarboxylase, as well as to increased polyamine uptake [6]. Several studies indicate that depletion of polyamines leads to inhibition of tumor growth [7,8,9].

A key role in regulating intracellular polyamine homeostasis is played by the ornithine decarboxylase enzyme, which is tightly regulated at multiple levels in normal and cancer cells, to promptly adjust the levels of polyamines in response to the specific cellular needs [10]. A first level of control of ODC content is through its stability. Indeed, ODC is a very short-lived protein and its rapid turnover is mediated by the proteasome in an ubiquitin-independent manner [11]. A protein called antizyme (AZ) associates with ODC and increases its targeting to the proteasome and consequent degradation [12]. Interestingly, AZ synthesis is induced by polyamines, leading to enhanced association to ODC monomers to form heterodimers and preventing the formation of functional ODC homodimers [11].

A second level of control of ODC content is trough its transcriptional regulation. ODC mRNA levels are regulated by various cues and transcription factors, being the oncogene MYC the best-characterized and more relevant regulator [13,14]. Indeed, the promoter region of the *Odc* gene contains two canonical E boxes (CACGTG) that bind MYC/Max transcription factors. Consistently, increased ODC expression is observed when MYC is upregulated, such as in cancer [15,16]. A third level of control of ODC expression is via its translation. The ODC mRNA has a long 5′ untranslated region (UTR) of about 300 nucleotides and is enhanced by elevated levels of eIF-4E [17], which binds the cap structure to initiate translation. Alternatively, ODC can be translated independently of cap-mediated initiation, using an internal ribosome entry site (IRES) located in the 5′ UTR [18]. This site would be used only under certain conditions such as in the G2/M phase of the cell cycle, or in response to developmental stimuli (see below).

Both ODC and AZ play an important role in carcinogenesis, as documented by studies in animal models. Targeted expression of an active C-terminally truncated form of ODC, under the control of keratin promoter significantly increased skin tumor development in mice treated with carcinogens or UV radiation or expressing active Ras [19,20,21,22]. Conversely, mice heterozygous for *Odc* gene (*Odc* +/−) developed substantially fewer skin papillomas when treated with a tumor-promoting agent [22]. Carcinogenesis was also reduced in mice expressing AZ under the keratin promoter and exposed to chemical of physical carcinogens [23], thus underscoring the relevance of ODC expression during skin carcinogenesis. In addition to skin tumors, Odc haploinsufficiency has been shown to significantly reduce Myc-induced lymphoma development in *Eμ-Myc* transgenic *Odc* +/− mice [24].

In agreement with these results, the use of the specific ODC inhibitor, DFMO (d,l-alpha-difluoromethylornithine), led to tumor reduction in animal models of different tumors [25].

Another key regulator of polyamine metabolism with relevance in tumor disease is the SAMDC enzyme, which catalyzes the decarboxylation of S-Adenosylmethionine (SAM) into decarboxylated SAM (dc-SAM). Dc-SAM is the aminopropyl donor for the synthesis of spermidine and sperimine, catalyzed by SpdS and SpmS respectively (Figure 1). SAMDC has been recently found upregulated by mTORC1 in prostate cancer via phosphorylation-mediated stabilization, thus providing an important link between the oncogenic nutrient-sensing machinery and polyamine metabolism and suggesting the potential therapeutic benefit of its targeting [26].

Given the role of the natural polyamines in cancer and growth-related processes, great efforts have been made to synthesize inhibitors for the enzymes involved in polyamine biosynthesis: spermidine and spermine synthase [27] ornithine decarboxylase [28] and S-adenosyl-methionine decarboxylase [29].

Strategies for cancer treatment are currently under development using:

1. Inhibitors of polyamine synthesis: (i) DFMO, a specific inhibitor of ornithine decarboxylase; currently, DMFO has been clinically tested in gliomas, neuroblastoma, colon, prostate and non melanoma skin cancer (NMSC, see below) [30]. (ii) methylglyoxal-bis-guanidylhydrazone (MGBG), an inhibitor of S-adenosyl-methionine decarboxylase [3], which reduces spermidine and spermine levels but elevates putrescine levels [31]. Although MGBG is an effective SAMDC inhibitor, its use in chemotherapy is restricted because of its mitochondrial toxicity [4]. (iii) SAM486A (4-amidinoindan-1-one-2′-amidinhydrazone) a derivative of MGBG. Despite it was tested in various cancer cells and animal systems, as well as in phase I and II clinical trials for activity against adult cancers, it resulted ineffective [31] probably because of the induction of compensatory mechanisms, which preserve the intracellular concentrations of polyamines [7].
2. Analogues of polyamines [32] which can deplete polyamine content and interfere with polyamine metabolism and/or function.
3. Polyamine transport inhibitors which can prevent uptake of exogenous polyamines by blocking membrane transporters [33].
4. Polyamine-degrading enzymes such as bovine serum amine oxidase (BSAO: EC 1.4.3.6) [34]. It was observed that the oxidative deamination of spermine by BSAO (bovine serum amine oxidase) generates ammonia and the cytotoxic metabolites hydrogen peroxide and aldehydes. Formation of cytotoxic aldehydes from polyamines or reactive oxygen species (ROS) may have potential in cancer therapy, in analogy to other radical forming processes [35], since these molecules are able to induce stress-activated signal transduction pathways, leading to apoptotic and non-apoptotic cell death, in several cultured tumor cell lines [36]. It has previously been demonstrated that hydrogen peroxide and aldehydes generated by BSAO/spermine enzymatic system were also able to overcome multidrug resistance (MDR) in cancer cells [37]. Therefore, toxic polyamine metabolites are currently explored as probable candidates for a new strategy in tumor therapy [35].

## 2. Hedgehog-Signaling and Its Targeting in Cancer

Hedgehog signaling regulates embryonic development and stem cell fate and its inappropriate activation causes different forms of cancer [38].

**Figure 2:**
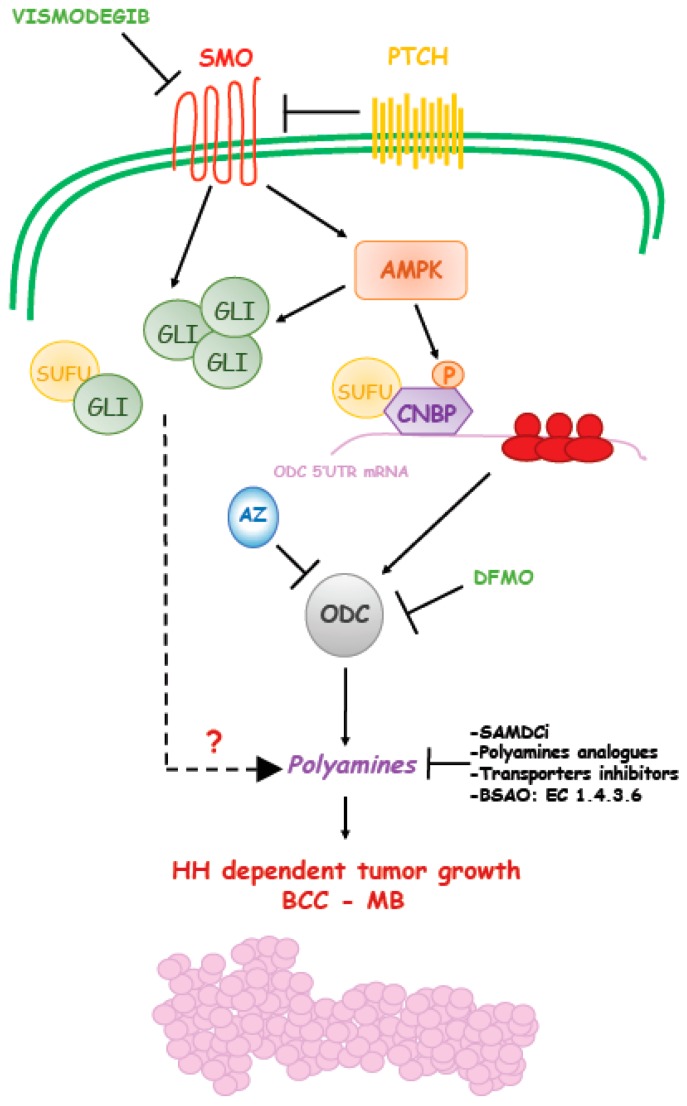
Schematic representation of Hedgehog-induced polyamines production during cancer formation. Hedgehog signaling induces polyamines levels through upregulation of ODC translation.

Transmembrane receptors and post-receptor proteins mediate the signal transduction, a process where the primary cilium plays a central role [39]. The receptor apparatus consists in (i) the inhibitory receptor patched (PTCH); and (ii) the transmembrane activator Smoothened (SMO). At the post-receptor level, the cytoplasmic regulator SUFU and the GLI transcription factors (GLI1, GLI2, GLI3) are the key mediators of the execution of the Hh transcriptional program [40] (Figure 2).

Germline loss-of-function mutations of the *PTCH* gene are found in the Gorlin syndrome, a genetic disease characterized by multiple skin tumors named Basal Cell Carcinoma (BCC) and, with lower frequency, by the brain tumors medulloblastoma (MB) [41].

Sporadic mutations of *PTCH1* or other genes encoding Hh pathway components, such as *SMO*, *SUFU*, and *GLI2*, can be found in nearly all BCCs [42,43] and in about one third of MBs (SHH-MB subgroup) [44,45,46] with various frequency (Table 1). In all cases, the overall consequence of these mutations is the constitutive activation of the Hh pathway, which thus represents a key tumorigenic driver for those types of malignancies.

The Hedgehog pathway can be targeted with drugs acting at different levels. Vismodegib has been the first FDA-approved Hh inhibitor and acts by inhibiting the Hh transmembrane activator Smoothened (SMO) [50]. However, despite the good efficacy observed in a subset of patients, results obtained with Vismodegib have been disappointing for two main reasons: (1) the drug is not effective in patients carrying mutations localized downstream of SMO [51,52]; (2) Even in patients that showed good response, tumor cells eventually become drug resistant, due to novel SMO mutations or activation of compensatory mechanisms that restore the function of the downstream effectors [38,53,54,55]. For these reasons, the identification of novel drugs targeting post-receptor Hh components is now a major goal in these malignancies. Several compounds with the ability to directly inhibit GLI transcription factors have been identified, but their efficacy, specificity and safety are still major issues that prevent their immediate use in clinical settings [56]. Also, since GLI activity is modulated by various post-translational modifications, another potential approach would be to use drugs with the ability to affect the different GLI modifiers, such as class I histone deacetylases (HDACs) [57,58,59,60,61,62,63], AMPK [64,65], ERK [66], MEKK1 [67], PIN1 [68].

An alternative strategy would be to target actionable Hh-regulated pathways or metabolisms that are required for tumor growth, using drugs of proven clinical efficacy and safety [38,69]. Among the various options, polyamine metabolism offers a reasonable therapeutic opportunity.

## 3. Targeting Polyamine Metabolism in Basal Cell Carcinoma

Basal cell carcinoma (BCC) is the most frequent tumor in humans, affecting about 3% of the population [70]. BCC rarely results in death but is often locally destructive, representing a significant economic burden to the health care system [71].

The lesion is typically treated with surgical excision, although there is a significant risk of recurrence. Metastasis occurs rarely, in 0.003–0.1% of patients [72].

The major risk factor of BCC is the prolonged exposure to UV light; hence the main preventive strategy is the reduction of sun exposure and the use of sunscreens. However, despite the risk associated to UV exposure is well known between the population, the incidence of BCC is still increasing over the time, thus raising the interest on the identification of additional preventive strategies [73,74].

Nearly all BCCs display aberrant activation of the Sonic Hedgehog pathway, due to loss of function mutations of PTCH [42,75] or activating mutations of SMO [76] and the Hedgehog inhibitor Vismodegib is approved for the treatment of locally advanced BCC (LaBCC) or metastatic BCC (mBCC) [77]. However, less than 50% of patients with advanced or metastatic BCCs respond to Vismodegib, and an additional 20% acquires secondary resistance during the first year of treatment [77,78,79]. Of note, drug-resistant tumors in patients maintain activation of Hedgehog target genes [55], witnessing an unchanged addiction to the Hedgehog pathway, due to activation of compensatory mechanisms that restore GLI function.

The adverse effects associated with Vismodegib are mild to moderate and include nausea, vomiting, diarrhea, constipation, muscle spasms, fatigue, hair loss, and dysgeusia (distortion of the sense of taste). Thus, the use of Vismodegib for treatment or prevention of BCC raises some legitimate concerns due to the significant risk to develop drug-resistance and the severity of the side effects, especially in case of long-term treatments.

Another key molecular alteration underlying BCC tumorigenesis is an increase of intracellular polyamines, mostly as a consequence of ODC overexpression. Early studies demonstrated that ODC activity is elevated in BCC, compared to normal tissues [80]. It was also shown that tumor promoting agents such as TPA [81] or UV light [82] induce ODC and polyamines, while ODC inhibition significantly limits skin tumor promotion [83].

The first preclinical evidence of a beneficial effect of targeting polyamine metabolism in Hh-induced BCC was documented in mice carrying heterozygous deletion of the *Ptch1* gene (*Ptch1*^+/−^). When exposed to UVB, these mice develop multiple and severe BCCs with high penetrance by the 20th week of irradiation. Tang et al. [23] demonstrated the importance of ODC and polyamines in this context. They observed that K6 promoter-driven expression of antizyme (AZ), a protein that specifically degrades ODC, in UVB-exposed *Ptch1*^+/−^ mice prevented BCC formation. Similarly, oral administration of DFMO to UVB-induced *Ptch1*^+/−^ mice strongly reduced the number and severity of the skin lesions. Thus, these observations demonstrated the relevance of polyamine metabolism in the pathogenesis of Hh-dependent BCC and provided a compelling preclinical evidence of the potential benefit that could result from the inhibition of a key polyamine controller, ODC, with a drug of known clinical efficacy and safety [23].

Following this observation, a few years later it was reported a phase III study on the cancer prevention ability of DFMO in patients with previous history of skin cancer. By evaluating a group of patients with a history of prior non-melanoma skin cancer (NMSC), randomized to 500 mg/m^2^/day oral DFMO or placebo for 4/5 years, Bailey and colleagues reported the first successful clinical trial of BCC prevention. DFMO significantly reduced new BCC, was well tolerated, with evidence of mild ototoxicity [71,84]. This study was updated two years later, retrospectively assessing the further incidence of skin cancer and adverse events in the same study cohort. The results showed that up to five years of DFMO treatment did not result in latent toxicity or in a rebound increase in rate of BCC after stopping DFMO [85]. Further studies would be appropriate to evaluate the effect of DFMO in combination with other agents.

## 4. Targeting Polyamine Metabolism in Medulloblastoma

Medulloblastoma represents the most common brain malignancy of the childhood, accounting for almost 20% of all cancers in children aged 0–19 [86]. The current therapeutic approach, based on a combination of surgery, craniospinal radiation and chemotherapy allows about 70% of survivors [87,88], although the majority of patients develop severe long-term side effects, mostly neurological [89,90,91].

Molecular analyses have identified four main molecular subgroups of MB, based on gene expression profiles: WNT, SHH, G3 and G4 [92,93,94]. Tumors of the SHH subgroup (SHH MB) show aberrant activation of the Hh signaling and account for about one third of total MBs. Similar to BCC, they are vulnerable to Hh inhibitors, although trials in patients have shown that they become quickly resistant to Vismodegib. Also, SHH MBs with mutations of *SUFU* or amplification of *GLI2* do not respond at all to this drug [95]. Thus, also for this malignancy there is an emerging need to identify additional drugs targeting post-receptor components of the signaling or regulated metabolic steps. In contrast to BCC, the correlation between SHH MB and polyamine metabolism is less documented. An old report by Scalabrino et al. identified an association between elevated ODC activity and medulloblastoma [96]. However, the distinction of MBs into various molecular subgroups was unknown at that time.

A recent paper showed the correlation of elevated ODC and polyamines with SHH MB molecular subgroup and provided the mechanistic basis of this alteration and its link with Hedgehog signaling [97]. It was observed that SMO activation triggers a non-canonical route that engages the energy sensor AMPK. The key mediator of this response is an RNA binding protein, named CNBP (Cellular Nucleic acid Binding Protein). AMPK phosphorylates CNBP at threonine 173 and this modification increases its association with SUFU and stabilization. The SUFU/CNBP complex binds the 5′ UTR of ODC mRNA and promotes its translation, thereby increasing polyamine metabolism. Thus, SMO activation promotes ODC translation via CNBP. This Hh-regulated translational process is required to support the proliferation of neuronal precursors and it is aberrantly activated in SHH MB, a tumor arising from these cells. Of note, in analogy to what was observed in BCC, targeting this process with the ODC inhibitor DFMO efficiently counteracts the growth of SHH MB in preclinical models. Hence, this report unveiled a novel druggable mechanism of the Hh signaling, where AMPK functions as a key tumor-promoting kinase, which regulates polyamine metabolism and supports tumor growth [97,98] (Figure 2). As a consequence, it would be expected that AMPK blockade would result in inhibition of this non-canonical Hedgehog signaling as well as inhibition of ODC translation and polyamine metabolism and suppression of tumor growth.

In keeping with this evidence, a very recent report showed that the lack of AMPKα2 in mouse cerebellum limits tumorigenesis of SHH MB through downregulation of CNBP and ODC [99]. Thus, in contrast to other reports [65,100,101] it was found that AMPK promotes SHH MB growth in mice, thus raising concerns about the use of agonists of this kinase for therapeutic purposes. Of note, in the same report the authors also found a copy number gain of the genes encoding CNBP and ODC, further supporting the pro-tumorigenic role of this non-canonical axis in SHH MB.

In contrast to BCC, clinical evidence on the efficacy of DFMO on SHH MB patients are still unavailable. A clinical trial of DFMO as a single agent on subjects with relapsed rare tumors, including medulloblastoma, has recently started (ClinicalTrials.gov identifier (NCT number: NCT03581240). Results from this study will provide critical information to understand the efficacy of the polyamine blockade to treat this type of tumor.

## Figures and Tables

**Table 1:** Frequency of mutations in Hh-dependent MB and BCC.

	Mutations in MB (Pediatric)	Mutations in BCC (Adults)
PTCH1	45% *	73% ***
SMO	10% *	20% ***
SUFU	35% *	8% ***
GLI2 amplification	7.3% **	8% ***
MYC-N amplification	12.7% **	30% ***

* [47]; ** [48]; *** [49].

## Abbreviations

Smo: Smoothened; Ptch: Patched; GLI: Glioma-associated oncogene; SUFU: Suppressor of Fused; SHH MB: Sonic Hedgehog type medulloblastoma; HDAC: Histone deacetylase; AMPK: 5′ adenosine monophosphate-activated protein kinase; ERK: extracellular signal–regulated kinases; MEKK1: Mitogen-activated protein kinase kinase kinase; TPA: tetradecanoyl phorbol acetate; WNT: Wingless/Integrated.
